# Predictors of dysfunction in fully versus partially covered self-expanding metal stents for malignant esophageal strictures: a 10-year multicenter study

**DOI:** 10.1186/s12876-026-05414-z

**Published:** 2026-09-30

**Authors:** Özlem Çelebi, Christian Jürgensen, Michael Sigal, Andreas Adler, Ian Levenfus, Konstantinos Kouladouros, Martin Maibier, Tobias Blasberg, Jürgen Hochberger, Frank Tacke, Tobias Kleemann, Quang Trung Tran, Marcel Razpotnik

**Affiliations:** 1 Department of Hepatology and Gastroenterology, Charité - Universitätsmedizin Berlin, Campus Virchow-Klinikum (CVK) and Campus Charité Mitte (CCM), Berlin, Germany; 2 Medical University of Lausitz – Carl Thiem, Cottbus, Germany

**Keywords:** Dysphagia, Esophageal Cancer, Esophageal Stricture, Self-Expanding Metal Stents, Stent Dysfunction

## Abstract

**Background:**

Esophageal self-expanding metal stents (SEMS) effectively restore oral intake in malignant obstruction, yet stent dysfunction remains a frequent clinical challenge. We aimed to identify and compare predictors of stent dysfunction in fully versus partially covered self-expanding metal stents (fcSEMS vs. pcSEMS).

**Methods:**

This multicenter retrospective cohort included patients undergoing SEMS placement for malignant esophageal strictures between 2014 and 2023. Stent dysfunction comprised migration, tumor overgrowth, or food bolus impaction. Patients with benign or anastomotic strictures, prior ablative therapy, or uncovered SEMS were excluded. Cumulative incidence and Fine-Gray subdistribution hazard models were used, treating death as a competing event. Interaction terms assessed effect modification by stent type.

**Results:**

Of 249 screened patients, 188 were included (mean age 68.0 ± 10.7 years; 78.7% male; 92 pcSEMS and 96 fcSEMS); technical success was 97.3%. Dysfunction occurred in 29.8% and was more frequent with fcSEMS than pcSEMS (36.5% vs. 22.8%; *p* = 0.04), primarily due to migration (31.3% vs. 10.9%; *p* < 0.01). Within 12 months, 37 patients (19.7%) died before dysfunction and were treated as competing events. In adjusted Fine-Gray models, strictures caused by other malignancies (non-squamous/non-adenocarcinoma) were strongly associated with dysfunction in the fcSEMS group (sHR 6.53; 95% CI 2.51–16.98; *p* < 0.01), with significant effect modification by stent type (sHR interaction 14.1; 95% CI 2.08–95.8; *p* < 0.01). Larger stricture diameter independently predicted dysfunction with fcSEMS (sHR per mm 1.21; 95% CI 1.01–1.45; *p* = 0.04), while lower esophageal location showed a borderline association (sHR 2.33; 95% CI 0.89–6.07; *p* = 0.08). Adverse-event rates were comparable (35.9% vs. 43.8%; *p* = 0.27).

**Conclusion:**

Patterns of stent dysfunction differed between fcSEMS and pcSEMS. Tumor etiology and stricture anatomy may guide risk-stratified stent selection and help reduce reinterventions.

**Trial registration:**

Not applicable.

## Introduction

Malignant esophageal strictures remain a major clinical challenge in therapeutic endoscopy, as many patients present with advanced-stage disease at diagnosis [1, 2]. They most commonly result from carcinomas of the distal esophagus or gastroesophageal junction (GEJ). Over recent decades, the epidemiology of esophageal cancer has shifted, with declining incidence of esophageal squamous cell carcinoma (SCC) and rising rates of esophageal adenocarcinoma (AC) in Western countries, likely reflecting changes in risk factors, including Barrett’s esophagus, gastroesophageal reflux disease, obesity, and tobacco use [3]. The overall prognosis for esophageal cancer remains poor, with 5-year survival rates typically below 30% [4].

Progressive dysphagia is the predominant presenting symptom and is often associated with worsening malnutrition and a marked decline in quality of life [5]. In this setting, endoscopic placement of self-expanding metal stents (SEMS) plays a central role in the palliation of malignant dysphagia, by restoring luminal patency and enabling early resumption of oral intake [6].

Despite ongoing technical improvements, stent dysfunction due to tumor overgrowth, migration, or food impaction still occurs in approximately 30% of patients [7, 8], often requiring repeat endoscopic interventions and increased healthcare utilization. Stent covering is presumed to affect both migration and tissue or tumor overgrowth; however, available studies have reported inconsistent findings regarding its impact on overall stent dysfunction. Beyond stent covering, reported risk factors for stent dysfunction are heterogeneous and include patient sex, tumor location, stent diameter, stricture traversability, and prior chemotherapy [8–10].

Evidence comparing cumulative incidence and time to stent dysfunction and its associated risk factors by stent covering remains limited. Previous studies have primarily examined overall predictors of stent dysfunction, and it remains unclear whether these associations differ between stent types. Defining stent type-specific predictors may enable more individualized SEMS selection and follow-up strategies, reduce reinterventions, and improve the management of malignant dysphagia. We therefore aimed to identify predictors of stent dysfunction within each stent type and formally assess whether their associations differed between partially covered (pcSEMS) and fully covered SEMS (fcSEMS).

## Methods

### Study design and population

**Fig. 1:**
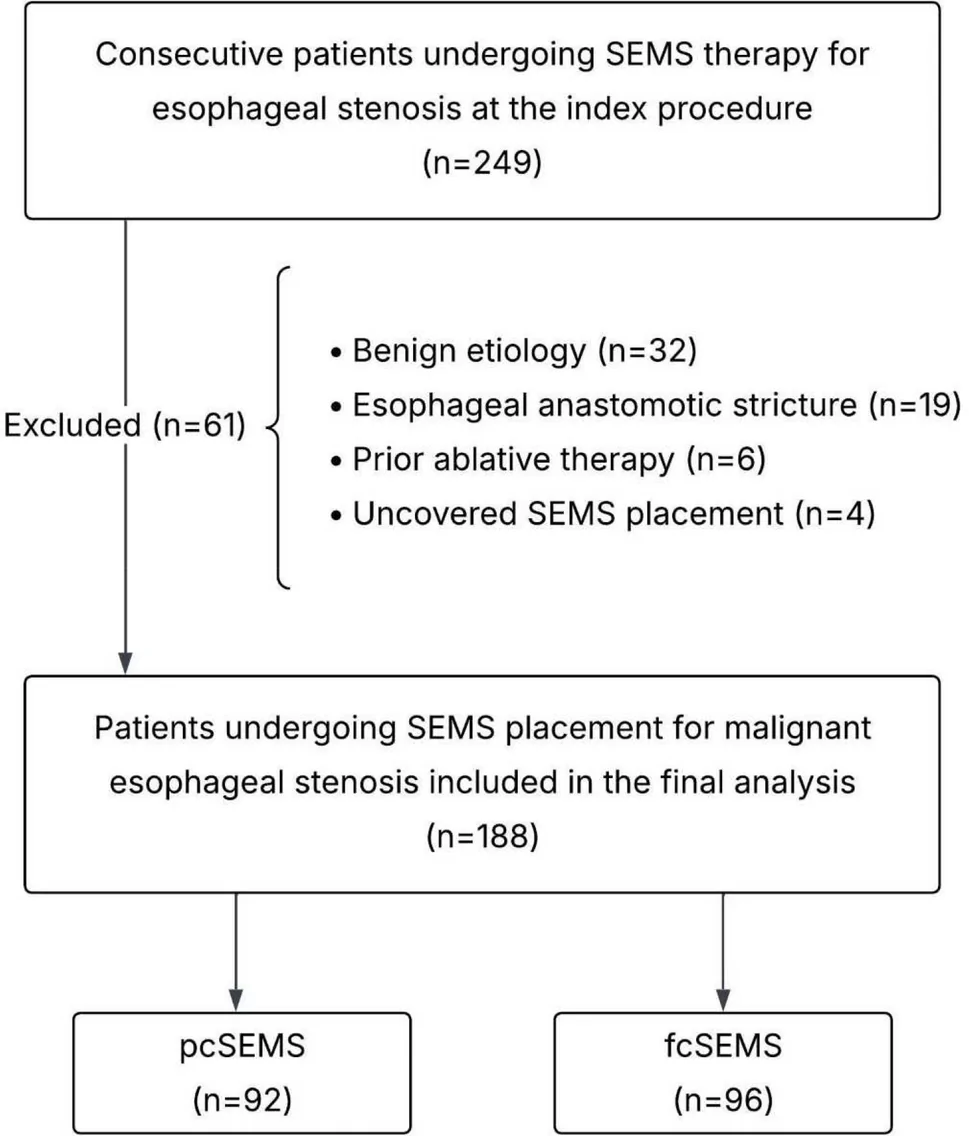
Flow diagram of patient selection showing inclusion and exclusion criteria. Abbreviations: fcSEMS, fully covered self-expanding metal stent; pcSEMS, partially covered self-expanding metal stent; SEMS, self-expanding metal stent

This multicenter retrospective study was performed at three tertiary academic centers between January 2014 and December 2023. All consecutive adults (≥ 18 years) undergoing endoscopic SEMS placement at the index procedure for a malignant esophageal stricture were eligible. Patients were excluded if: (1) stent placement was performed for indications other than restoration of luminal patency in malignant strictures, (2) prior endoscopic ablative therapy had been performed, (3) recurrent carcinoma at the esophageal anastomosis was present, or (4) an uncovered SEMS was used (Fig. 1).

Data were collected by experienced physicians following a predefined protocol. Information was retrieved from electronic health records, including endoscopy databases and radiological reports. Variables included demographic and clinical patient characteristics, stricture-related parameters, and procedural factors.

The study was conducted in accordance with the Declaration of Helsinki and approved by the institutional review board of Charité-Universitätsmedizin Berlin (reference number: EA1/120/25); informed consent was waived because of the retrospective design.

### Outcomes and definitions

The primary outcome was the cumulative incidence of stent dysfunction at predefined time points after SEMS placement, compared between stent types. Stent dysfunction was defined as the failure to maintain luminal patency resulting from stent migration, tumor or tissue ingrowth or overgrowth, food bolus impaction, or other stent-related causes [7, 8, 11]. Patients were followed until stent dysfunction, death, stent removal, last documented follow-up, or administrative censoring at 12 months.

Secondary outcomes included technical success, defined as successful stent deployment with correct positioning across the stricture, the total number of endoscopic procedures, and adverse events, with severity graded according to the American Society for Gastrointestinal Endoscopy (ASGE) lexicon [12].

The esophagus was divided into upper (16–23 cm), middle (24–31 cm), and lower (32–40 cm) thirds based on the distance from the incisors. Stricture location was defined by the distance from the incisors to the epicenter of the stricture.

Dysphagia severity was assessed using the modified Dakkak and Ogilvie scales, according to patient-reported ability to swallow different food consistencies (Dakkak: 0, no dysphagia; 1, dysphagia to solids; 2, dysphagia to semisolids; 3, dysphagia to liquids; 4, aphagia; Ogilvie: 0, normal diet; 1, some solids; 2, semisolids only; 3, liquids only; 4, complete dysphagia, including liquids) [13, 14].

### Endoscopic procedure and SEMS placement

Malignant strictures were first evaluated endoscopically. Whenever feasible, the stricture was traversed to allow complete examination of the upper gastrointestinal tract. If the stricture could not be passed with a standard gastroscope (Olympus GIF-H190 or Olympus GIF-HQ190, Olympus Medical Systems, Tokyo, Japan), a pediatric gastroscope (Olympus GIF-XP190N) was typically used. SEMS placement was performed over a guidewire under endoscopic guidance, with or without fluoroscopy. Additional stent fixation was not routinely performed across centers.

The choice of stent type was made at the discretion of the endoscopist and local availability of stent models. The following SEMS were predominantly used: WallFlex (Boston Scientific, Marlborough, MA, USA; fully and partially covered), Ultraflex (Boston Scientific; partially covered), Hanaro stents (M.I. Tech, Pyeongtaek, South Korea; fully covered), AIX (Leufen Medical, Berlin, Germany; fully and partially covered), and Micro-Tech esophageal stents (Micro-Tech, Nanjing, China; fully and partially covered). Other models were used only in a small number of cases (*n* = 8).

If incorrect stent positioning was clinically suspected immediately after the procedure (≤ 24 h), plain radiography or upper endoscopy was performed. These cases were classified as misdeployment and analyzed as technical failures. Subsequent stent dysfunction analyses were based exclusively on the successfully placed replacement stent.

### Statistical analysis

The normality of continuous variables was assessed using the Shapiro-Wilk test. Continuous variables were presented as means ± standard deviations (SDs) or medians with interquartile ranges (IQRs), as appropriate. Categorical variables were reported as counts and percentages. Comparisons between groups were performed using the Chi-square test or Fisher’s exact test for categorical variables and the Student’s t-test or Mann-Whitney U test for continuous variables, based on data distribution.

Stent dysfunction at 3, 6, and 12 months was analyzed using Fine-Gray competing-risk models, with death treated as a competing event [15]. Cumulative incidence functions were compared using Gray’s test [16]. Associations with stent dysfunction were reported as subdistribution hazard ratios (sHRs) with 95% confidence intervals (CIs) and adjusted for age, sex, body mass index (BMI), American Society of Anesthesiologists (ASA) class, and prior chemoradiotherapy. These covariates were selected based on clinical relevance supported by previous literature and their potential confounding effects, while limiting model complexity to reduce overfitting.

Separate interaction models assessed whether the association between each candidate predictor and stent dysfunction differed by stent type. These models included the candidate predictor (X), stent type (S; pcSEMS = 0 [reference], fcSEMS = 1), and a predictor-stent type interaction term (XS). A significant interaction term indicated that the association with the subdistribution hazard of stent dysfunction differed between fcSEMS and pcSEMS [17]. Statistical analyses were performed using R software (v. 4.6.1). Figures were generated in Python (v. 3.11.7; Anaconda, Inc.) using the open-source Matplotlib library. A two-sided p-value < 0.05 was considered statistically significant.

## Results

### Cohort characteristics

A total of 188 patients who underwent SEMS placement at the index procedure for malignant esophageal strictures were included in the final analysis. The mean age was 68 ± 10.7 years, and 148 patients (78.7%) were male. Esophageal SCC was the most common etiology (79/188, 42.0%), followed by esophageal AC (69/188, 36.7%) and other malignancies (39/188, 20.7%); histology was unspecified in one patient. Among the 39 patients with other malignancies, 26 had intrinsic or infiltrative tumor involvement, including 11 rare primary esophageal histological subtypes, 4 non-cardia gastric cancers, and 11 cases of direct esophageal wall infiltration from lung or other metastatic malignancies. Extrinsic compression was documented in 11 patients, while the underlying mechanism remained indeterminate in 2. The majority of tumors were located in the distal esophagus (119/188, 63.3%).

Comorbidities were present in 152 patients (80.9%), most commonly cardiac disease (*n* = 82, 43.6%), followed by pulmonary disease (*n* = 42, 22.3%), diabetes mellitus (*n* = 33, 17.6%), renal disease (*n* = 20, 10.6%), and hepatic disease (*n* = 11, 5.9%). Patients receiving pcSEMS had a higher proportion of ASA class ≥ III (68.5% vs. 42.7%; *p* < 0.01). In contrast, fcSEMS were more frequently used in SCC (55.3% vs. 28.3%; *p* < 0.01) and in shorter strictures (median 50 mm vs. 65 mm; *p* = 0.04). A detailed baseline comparison is provided in Table 1.

**Table 1:** Baseline characteristics

	All SEMS	pcSEMS	fcSEMS	*P* ^a^
	*n* = 188	*n* = 92	*n* = 96	
**Patient characteristics**				
Age, years, mean (SD)	68 ± 10.7	67.1 ± 11.6	68.9 ± 9.9	0.257
Sex, male, n (%)	148 (78.7)	68 (73.9)	80 (83.3)	0.115
BMI, kg/m², median (IQR)	22 (19–25)	22 (19–25)	22.5 (20–25)	0.535
**Comorbidity and baseline status**				
Comorbidities, all, n (%)	152 (80.9)	72 (78.3)	80 (83.3)	0.377
Cardiac, n (%)	82 (43.6)	38 (41.3)	44 (45.8)	0.531
Pulmonary, n (%)	42 (22.3)	28 (30.4)	14 (14.6)	0.009
Hepatic, n (%)	11 (5.9)	5 (5.4)	6 (6.3)	0.812
Renal, n (%)	20 (10.6)	15 (16.3)	5 (5.2)	0.014
Diabetes mellitus, n (%)	33 (17.6)	17 (18.5)	16 (16.7)	0.744
ASA class ≥ III, n (%)	104 (55.3)	63 (68.5)	41 (42.7)	< 0.001
KPS, %, median (IQR)	80 (70–90)	80 (60–80)	80 (70–90)	< 0.001
**Etiology**				
Adenocarcinoma, n (%)	69 (36.7)	35 (38)	34 (35.4)	0.749
Squamous cell carcinoma, n (%)	79 (42.0)	26 (28.3)	53 (55.2)	< 0.001
Other malignancies, n (%)	39 (20.7)	31 (33.7)	8 (8.3)	< 0.001
**Prior/concomitant therapy**				
Prior chemoradiotherapy, n (%)	34 (18.1)	21 (22.8)	13 (13.5)	0.105
Proton pump inhibitor, n (%)	142 (75.5)	69 (75.0)	73 (76.0)	0.870
Anticoagulant therapy, n (%)	32 (17.0)	15 (16.3)	17 (17.7)	0.799
Antiplatelet therapy, n (%)	51 (27.1)	27 (29.3)	24 (25.0)	0.501
**Dysphagia severity**				
Dakkak dysphagia score, median (IQR)	2 (1–2)	2 (1–2)	2 (1–2)	0.255
Ogilvie dysphagia score, median (IQR)	2 (2–3)	3 (2–3)	2 (2–3)	0.482
**Laboratory**				
Serum albumin, g/L, median (IQR)	35.5 (31.1–39.8)	35.3 (28.9–39.2)	36.0 (31.8–40.5)	0.319
Platelets, ×10⁹/L, median (IQR)	266 (206–325)	256 (177–334)	273 (213–319)	0.337
Prothrombin time activity, %, median (IQR)	89 (80–98)	87 (78–96)	90 (81–100)	0.164
**Procedure/technique**				
Pediatric gastroscope, n (%)	52 (27.7)	30 (32.6)	22 (22.9)	0.138
Passed endoscopically, n (%)	122 (64.9)	64 (69.6)	58 (60.4)	0.183
**Tumor location**				
Distance from incisors, cm, median (IQR)	30 (25–35)	30 (25–35)	30.5 (26.5–35)	0.674
Upper esophagus, n (%)	15 (8)	6 (6.5)	9 (9.4)	0.470
Middle esophagus, n (%)	54 (28.7)	30 (32.6)	24 (25)	0.249
Lower esophagus, n (%)	119 (63.3)	56 (60.9)	63 (65.6)	0.499
GEJ involved, n (%)	67 (35.6)	33 (35.9)	34 (35.4)	0.948
**Stricture/stent metrics**				
Stricture length, mm, median (IQR)	60 (40–90)	65 (50–100)	50 (40–80)	0.042
Stricture diameter, mm, median (IQR)	8 (6–9)	8 (6–9)	7 (6–9)	0.816
Stent length, mm, median (IQR)	105 (100–124)	105 (100–123)	103 (80–125)	0.065
Stent diameter, mm, median (IQR)	18 (18–23)	20 (18–23)	18 (18–18)	< 0.001

Abbreviations: ASA, American Society of Anesthesiologists; BMI, body mass index; fcSEMS, fully covered self-expanding metal stent; GEJ, gastroesophageal junction; IQR, interquartile range; KPS, Karnofsky Performance Status; pcSEMS, partially covered self-expanding metal stent; SEMS, self-expanding metal stent; SD, standard deviation

^a^ Chi-square test, Fisher’s exact test, Student’s t-test, or Mann-Whitney U test, as appropriate, was used for comparison between pcSEMS and fcSEMS

### Procedural characteristics and clinical outcomes

A total of 188 SEMS were deployed (92 pcSEMS and 96 fcSEMS), distributed across the three participating centers with 79, 35, and 74 cases, respectively. Technical success was 97.3% and did not differ by stent type. All five initial failures were successfully salvaged by repeat SEMS placement during the same procedure or later that day. Of 38 stent removals, 31 were attributable to stent dysfunction. The remaining seven were related to early misdeployment (*n* = 5) or severe pain (*n* = 2) and were not classified as dysfunction events.

The median number of endoscopic interventions was comparable between groups (2 vs. 2; *p* = 0.18). A pediatric gastroscope was required in 52 cases (27.7%). Stent length ranged from 60 to 155 mm, and 18-mm diameter was the most common stent size (61.7%), while 52 stents (27.7%) measured 23–24 mm. Dysfunction occurred in 10 of these large-diameter SEMS (19.2%), comprising migration in 5 (9.6%), tumor overgrowth in 4 (7.7%), and food bolus obstruction in 1 (1.9%). Compared with smaller stents, these large-diameter SEMS showed lower rates of overall dysfunction and migration (*p* = 0.04 and *p* = 0.01, respectively). However, stent diameter was not independently associated with dysfunction in the adjusted competing-risks model (< 23 mm vs. ≥23 mm; sHR 1.19; 95% CI 0.55–2.59; *p* = 0.66).

The overall adverse event rate was 39.9% (75/188) and did not differ between stent types (fcSEMS 43.8% vs. pcSEMS 35.9%; *p* = 0.27). Most adverse events were categorized as moderate in severity, and severe events were observed in 3.2% of patients, including three cases of bleeding and three cases of fistula formation (Table 2). One patient subsequently underwent surgery due to recurrent bleeding. The remaining five patients required intensive care admission and prolonged hospitalization following the endoscopic intervention, of whom two died due to recurrent bleeding. In both cases, progressive disease was present and no further intervention was pursued in accordance with patient preferences for palliative care.

**Table 2:** Technical and clinical outcomes according to the stent type

	All SEMS	pcSEMS	fcSEMS	*P* ^a^
	*n* = 188	*n* = 92	*n* = 96	
Technical success, n (%)	183 (97.3)	91 (98.9)	92 (95.8)	0.369
No. of all procedures, median (IQR)	2 (1–3)	2 (1–3)	2 (1–3)	0.175
Stent dysfunction, n (%)	56 (29.8)	21 (22.8)	35 (36.5)	0.041
Overgrowth/ingrowth, n (%)	12 (6.4)	8 (8.7)	4 (4.2)	0.204
Stent migration, n (%)	40 (21.3)	10 (10.9)	30 (31.3)	< 0.001
Food bolus, n (%)	2 (1.1)	1 (1.1)	1 (1)	1.000
Other stent dysfunction, n (%)	2 (1.1)	2 (2.2)	0 (0)	0.238
Stent removal, n (%)	38 (20.2)	14 (15.2)	24 (25)	0.095
Stent-in-stent, n (%)	21 (11.2)	11 (12)	10 (10.4)	0.738
Stent fixation, n (%)	8 (4.3)	1 (1.1)	7 (7.3)	0.065
Adverse events, n (%)	75 (39.9)	33 (35.9)	42 (43.8)	0.270
Bleeding, n (%)	5 (2.7)	4 (4.3)	1 (1)	0.204
Severe pain, n (%)	10 (5.3)	6 (6.5)	4 (4.2)	0.530
Fistula, n (%)	4 (2.1)	2 (2.2)	2 (2.1)	1.000
Mild AE, n (%)	5 (2.7)	3 (3.3)	2 (2.1)	0.678
Moderate AE, n (%)	64 (34.0)	26 (28.3)	38 (39.6)	0.101
Severe AE, n (%)	6 (3.2)	4 (4.3)	2 (2.1)	0.437
3-month mortality, n (%)	29 (15.4)	18 (19.6)	11 (11.5)	0.124
6-month mortality, n (%)	41 (21.8)	26 (28.3)	15 (15.6)	0.036
12-month mortality, n (%)	48 (25.5)	28 (30.4)	20 (20.8)	0.131

Abbreviations: AE, adverse event; IQR, interquartile range

^a^ Chi-square test, Fisher’s exact test or Mann-Whitney U test, as appropriate for comparison between pcSEMS and fcSEMS

Within 12 months of SEMS placement, 37 patients (19.7%) died before stent dysfunction and were treated as competing events in the Fine-Gray analysis: 23 patients in the pcSEMS group (25.0%) and 14 in the fcSEMS group (14.6%; *p* = 0.07).

### Stent dysfunction according to the manufacturer

Stent dysfunction rates differed across manufacturers, ranging from 20.7% to 41.2%, with a significant overall difference among the four main groups (*p* = 0.04). However, the distribution of fully and partially covered stents differed substantially across manufacturers, and some groups included only a small number of stents and dysfunction events (Table 3). Therefore, this analysis was considered exploratory, and the findings should be interpreted with caution.

**Table 3:** Stent distribution and dysfunction according to stent type and model

Stent model	Coverage	Number of Stents	Dysfunction	Migration
		*n* (% of all)		*n* (% of group)
WallFlex (Boston Scientific)	Fully	18 (9.6)	3 (16.7)	2 (11.1)
WallFlex (Boston Scientific)	Partially	23 (12.2)	3 (13)	2 (8.7)
Ultraflex (Boston Scientific)	Partially	41 (21.8)	11 (26.8)	5 (12.2)
Hanaro (M.I. Tech)	Fully	68 (36.2)	28 (41.2)	26 (38.2)
AIX (Leufen Medical)	Fully	1 (0.5)	0 (0)	0 (0)
AIX (Leufen Medical)	Partially	13 (6.9)	3 (23.1)	1 (7.7)
Micro-Tech esophageal stent	Fully	5 (2.7)	3 (60)	2 (40)
Micro-Tech esophageal stent	Partially	11 (5.9)	2 (18.2)	1 (9.1)
*Other rare stent models	Fully	4 (2.1)	1 (25)	0 (0)
*Other rare stent models	Partially	4 (2.1)	2 (50)	1 (25)

Percentages for individual stent models refer to the total cohort (*n* = 188), whereas dysfunction and migration percentages refer to the respective stent models. *Other rare stent models included ENDO-FLEX (ENDO-FLEX GmbH, Voerde, Germany; *n* = 4), Bonastent (Standard Sci-Tech Inc., Seoul, South Korea; *n* = 2), Niti-S (Taewoong Medical Co., Ltd., Gimpo-si, South Korea; *n* = 1), and Evolution (Cook Ireland Ltd., Limerick, Ireland; *n* = 1)

### Cumulative incidence of stent dysfunction

Stent dysfunction occurred more frequently with fcSEMS than with pcSEMS (36.5% vs. 22.8%; *p* = 0.04), largely driven by a higher migration rate (31.3% vs. 10.9%; *p* < 0.01). At 3, 6, and 12 months, the adjusted cumulative incidence of dysfunction was 22.2% (95% CI, 11.4–34.5), 32.1% (95% CI, 19.1–47.5), and 42.2% (95% CI, 24.8–60.5) for pcSEMS, compared with 28.8% (95% CI, 17.8–39.4), 42.7% (95% CI, 29.8–54.6), and 49.6% (95% CI, 35.2–64.1) for fcSEMS, respectively (Gray`s test, *p* = 0.23). Stent fixation was used in 7.3% of fcSEMS placements, with migration occurring in three of these cases.

**Fig. 2:**
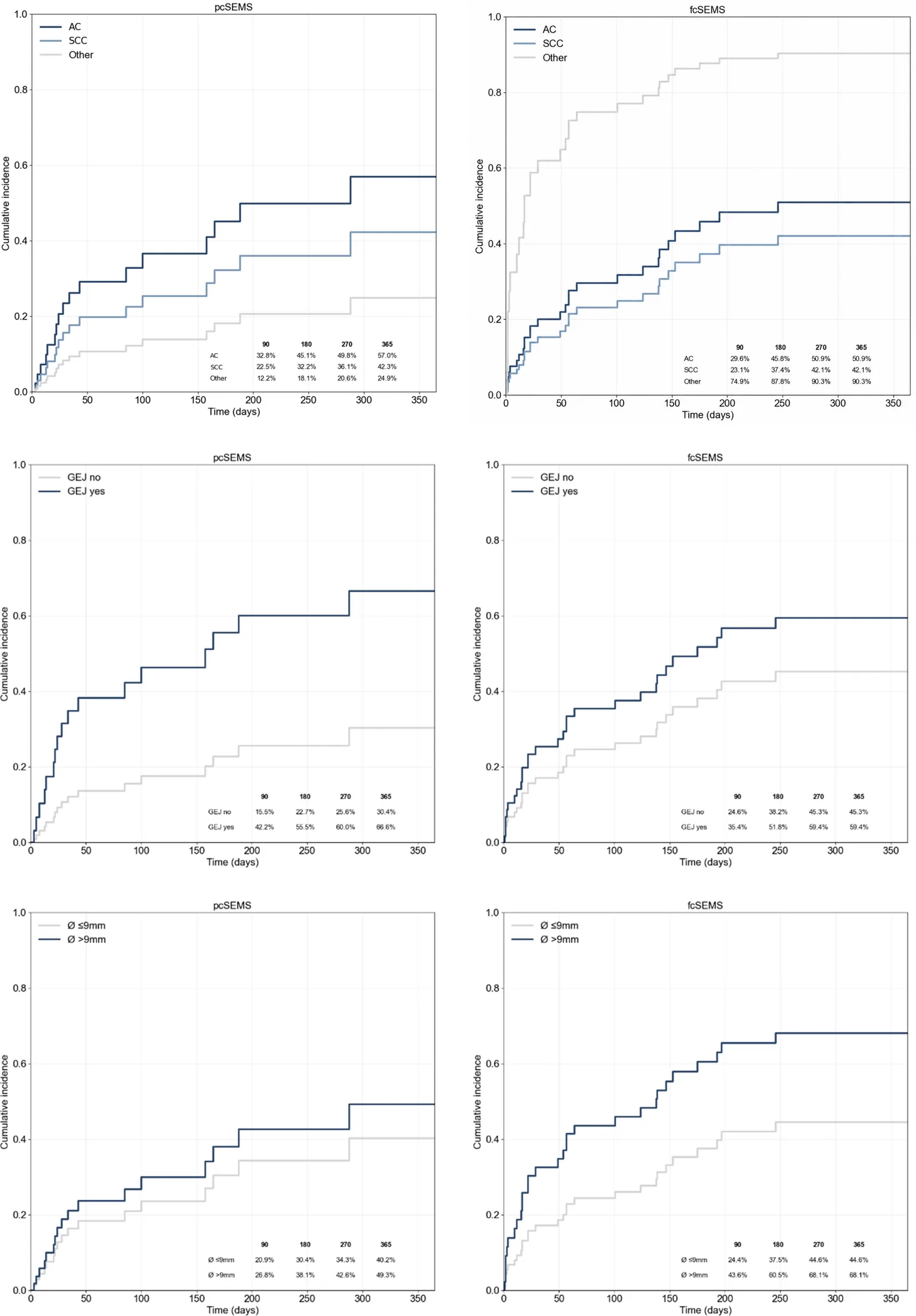
Adjusted cumulative incidence curves for stent dysfunction over time according to predictor group and stent type, with mortality treated as a competing risk. Abbreviations: AC, esophageal adenocarcinoma; fcSEMS, fully covered self-expanding metal stent; GEJ, gastroesophageal junction; pcSEMS, partially covered self-expanding metal stent; SCC, esophageal squamous cell carcinoma; Ø, stricture diameter

In the overall cohort, the cumulative incidence differed significantly according to the need for pediatric gastroscope use at the index procedure (*p* = 0.01), GEJ involvement (*p* = 0.04), and stricture diameter > 9 mm (*p* = 0.04). Adjusted cumulative incidence curves stratified by tumor etiology, GEJ involvement, and stricture diameter are presented for both stent groups in Fig. 2.

Among patients with GEJ involvement, the 3-month cumulative incidence of dysfunction was 42.2% with pcSEMS and 35.4% with fcSEMS, increasing at 12 months to 66.6% and 59.4%, respectively. In patients with strictures > 9 mm, the corresponding incidence at 3 months was lower with pcSEMS than with fcSEMS (26.8% vs. 43.6%).

Within the fcSEMS group, cumulative incidence differed significantly by etiology (*p* = 0.02). The 3-month incidence was highest in patients with strictures caused by other malignancies (74.9%), compared with adenocarcinoma (29.6%) and squamous cell carcinoma (23.1%). However, the small number of cases in the “other malignancies” subgroup limits the robustness of this estimate, which should therefore be interpreted as exploratory and hypothesis-generating. The corresponding incidences in the pcSEMS group were 12.2%, 32.8%, and 22.5%, respectively (Table 4).

**Table 4:** Cumulative incidence of stent dysfunction at fixed time points stratified by stent type, with mortality treated as a competing risk

	Cumulative incidence (%)^a^										
	pcSEMS				fcSEMS					*P* ^c^	
	3-month	6-month	12-month	*P* ^b^	3-month	6-month	12-month	*P* ^b^			
**Etiology**											
Adenocarcinoma	32.8	45.1	57.0	0.58	29.6	45.8	50.9	0.02		0.44	
Squamous cell carcinoma	22.5	32.2	42.3		23.1	37.4	42.1				
Other malignancies	12.2	18.1	24.9		74.9	87.8	90.3				
**Procedure/technique**											
Passed endoscopically (yes)	22.7	32.8	42.9	0.91	31.3	46.8	54.5	0.26		0.47	
Passed endoscopically (no)	21.1	30.5	40.2		22.4	35.2	42.1				
**Tumor location**											
Distance from incisors > 30 cm	25.3	36.1	46.8	0.96	36.9	54	62.2	0.13		0.09	
Distance from incisors ≤ 30 cm	20.2	29.3	38.8		19.8	32.2	39.2				
Upper esophagus	34.1	47.6	60.0	0.51	9.7	16.4	20.4	0.16		0.33	
Middle esophagus	21.1	30.9	41.0		21.4	33.8	40.5				
Lower esophagus	21.2	31	41.2		33.0	48.9	56.5				
GEJ involved (yes)	42.2	55.5	66.6	0.30	35.4	51.8	59.4	0.27		0.04	
GEJ involved (no)	15.5	22.7	30.4		24.6	38.2	45.3				
**Stricture/stent metrics**											
Stricture length > 60 mm	25.9	37.3	48.4	0.69	39.2	56.1	63.5	0.09		0.30	
Stricture length ≤ 60 mm	19.6	28.9	38.4		22.9	36.0	42.7				
Stricture diameter > 9 mm	26.8	38.1	49.3	0.63	43.6	60.5	68.1	0.07		0.04	
Stricture diameter ≤ 9 mm	20.9	30.4	40.2		24.4	37.5	44.6				
Stent length > 100 mm	15.3	22.8	30.3	0.24	29.2	43.3	50.2	0.99		0.28	
Stent length ≤ 100 mm	35.4	48.7	60.1		28.1	41.9	48.8				
Stent diameter > 20 mm	20.0	29.2	38.8	0.37	34.7	50.0	57.3	0.55		0.38	
Stent diameter ≤ 20 mm	25.1	36.0	47.0		27.7	41.4	48.3				

Abbreviations: fcSEMS, fully covered self-expanding metal stent; GEJ, gastroesophageal junction; pcSEMS, partially covered self-expanding metal stent

Cut-off values for the respective variables were defined using the mean value of the overall cohort, and patients below and above these thresholds were compared. ^a^ Cumulative incidence function adjusted for age, sex, BMI, ASA class, and prior chemoradiotherapy;

^b^Gray’s test comparing strata within each group; ^c^ Gray’s test comparing strata in the overall cohort

### Fine-Gray subdistribution hazard models and stent type interactions

In unadjusted Fine-Gray models, several clinical, tumor-related, and procedural factors were associated with stent dysfunction (Table 5). Significant interactions with stent type were observed for lower esophageal location (sHR interaction 4.66; 95% CI 1.36–15.9; *p* = 0.01).

**Table 5:** Unadjusted Fine-Gray subdistribution hazard models for stent dysfunction by stent type, with mortality treated as a competing risk

	pcSEMS		fcSEMS		Interaction (pcSEMS ref.)	
	sHR (95% CI)	*P*	sHR (95% CI)	*P*	sHR_int_ (95% CI)	*P*_int_
**Patient characteristics**						
Age, years	1.006 (0.954–1.061)	0.82	1.049 (1.018–1.081)	< 0.01	1.043 (0.982–1.108)	0.17
Sex, male	1.044 (0.323–3.371)	0.94	0.824 (0.300–2.259)	0.71	0.833 (0.176–3.947)	0.82
BMI, kg/m^2^	0.989 (0.928–1.054)	0.74	1.089 (1.002–1.183)	0.05	1.104 (0.990–1.231)	0.08
**Comorbidity and baseline status**						
Comorbidities, all	0.922 (0.313–2.718)	0.26	1.495 (0.682–3.278)	0.32	1.589 (0.415–6.083)	0.50
Cardiac	1.378 (0.570–3.336)	0.48	2.072 (1.064–4.037)	0.03	1.445 (0.472–4.425)	0.52
Pulmonary	1.143 (0.453–2.882)	0.77	0.908 (0.372–2.220)	0.83	0.791 (0.216–2.894)	0.72
Hepatic	0.664 (0.117–3.760)	0.64	1.966 (0.647–5.975)	0.23	2.887 (0.353–23.621)	0.32
Renal	1.180 (0.326–4.271)	0.80	1.293 (0.449–3.723)	0.63	1.054 (0.198–5.624)	0.95
Diabetes mellitus	1.355 (0.422–4.350)	0.61	2.390 (1.260–4.532)	< 0.01	1.745 (0.459–6.637)	0.41
ASA class ≥ III	1.504 (0.568–3.981)	0.41	1.493 (0.776–2.873)	0.23	1.007 (0.306–3.314)	0.99
KPS	0.992 (0.961–1.025)	0.65	0.996 (0.968–1.025)	0.79	1.003 (0.960–1.048)	0.89
**Etiology**						
Adenocarcinoma	1.417 (0.585–3.436)	0.44	2.087 (1.070–4.070)	0.03	1.417 (0.466–4.311)	0.54
Squamous cell carcinoma	1.019 (0.398–2.609)	0.97	0.382 (0.192–0.758)	< 0.01	0.381 (0.118–1.234)	0.11
Other malignancies	0.635 (0.205–1.969)	0.43	2.127 (0.727–6.223)	0.16	3.588 (0.732–17.587)	0.12
**Prior/concomitant therapy**						
Prior chemoradiotherapy	1.630 (0.596–4.460)	0.34	0.341 (0.086–1.356)	0.13	0.217 (0.040–1.194)	0.08
Proton pump inhibitor	1.929 (0.554–6.717)	0.30	1.287 (0.519–3.192)	0.59	0.662 (0.142–3.095)	0.60
Anticoagulation therapy	0.662 (0.166–2.645)	0.56	1.592 (0.761–3.328)	0.22	2.327 (0.475–11.390)	0.30
Antiplatelet therapy	1.100 (0.424–2.852)	0.85	1.514 (0.781–2.935)	0.22	1.360 (0.418–4.422)	0.61
**Dysphagia severity**						
Dakkak dysphagia score	1.020 (0.617–1.686)	0.94	0.772 (0.511–1.166)	0.22	0.761 (0.394–1.471)	0.42
Ogilvie dysphagia score	1.050 (0.589–1.870)	0.87	0.533 (0.290–0.978)	0.04	0.503 (0.211–1.198)	0.12
**Laboratory**						
Serum albumin, g/L	1.074 (0.995–1.158)	0.07	1.014 (0.964–1.067)	0.59	0.943 (0.856–1.040)	0.24
Platelets, ×10⁹/L	0.997 (0.994–1.001)	0.14	0.997 (0.992–1.002)	0.22	0.999 (0.993–1.005)	0.79
Prothrombin time activity, %	0.995 (0.961–1.031)	0.80	0.993 (0.974–1.013)	0.50	0.307 (0.003–29.377)	0.61
**Procedure/technique**						
Passed endoscopically	1.153 (0.419–3.173)	0.78	1.622 (0.822–3.200)	0.16	1.63 (0.47–5.74)	0.45
**Tumor location**						
Distance from incisors, cm	0.968 (0.891–1.052)	0.45	1.046 (0.998–1.098)	0.06	1.079 (0.980–1.188)	0.12
Upper esophagus	2.226 (0.484–10.236)	0.30	0.266 (0.037–1.894)	0.19	0.118 (0.010–1.424)	0.09
Middle esophagus	1.268 (0.512–3.139)	0.61	0.425 (0.174–1.035)	0.06	0.342 (0.095–1.234)	0.10
Lower esophagus	0.632 (0.259–1.546)	0.32	2.990 (1.298–6.886)	< 0.01	4.659 (1.360–15.967)	0.01
GEJ involved	1.553 (0.649–3.717)	0.32	1.908 (0.989–3.680)	0.05	1.197 (0.397–3.613)	0.75
**Stricture/stent metrics**						
Stricture length, mm	1.002 (0.990–1.015)	0.73	1.007 (0.995–1.018)	0.27	1.005 (0.987–1.022)	0.61
Stricture diameter, mm	1.062 (0.828–1.362)	0.64	1.189 (1.043–1.355)	< 0.01	1.126 (0.847–1.498)	0.41
Stent length, mm	0.997 (0.972–1.023)	0.82	0.999 (0.987–1.012)	0.93	1.002 (0.974–1.031)	0.87
Stent diameter, mm	0.898 (0.715–1.128)	0.35	1.012 (0.830–1.234)	0.91	1.200 (0.917–1.571)	0.18

Abbreviations: ASA, American Society of Anesthesiologists; BMI, body mass index; fcSEMS, fully covered self-expanding metal stent; GEJ, gastroesophageal junction; KPS, Karnofsky Performance Status; pcSEMS, partially covered self-expanding metal stent; *P*_int_, p value for interaction; *ref.*, reference; sHR, subdistribution hazard ratio; sHR_int_, interaction subdistribution hazard ratio

After adjustment, other malignancies showed the strongest association with dysfunction in the fcSEMS compared with the pcSEMS group (adjusted sHR 6.53; 95% CI 2.51–16.9; *p* < 0.01 vs. 0.41; 95% CI 0.11–1.55; *p* = 0.19), with significant effect modification by stent type (sHR interaction 14.1; 95% CI 2.08–95.8; *p* < 0.01).

Larger stricture diameter independently predicted stent dysfunction in the fcSEMS group, with each 1-mm increase associated with a 21% higher subdistribution hazard (adjusted sHR 1.21; 95% CI 1.01–1.45; *p* = 0.04). No corresponding association was observed in the pcSEMS group (adjusted sHR 0.99; *p* = 0.92).

In the fcSEMS group, the Ogilvie dysphagia score, distance from the incisors, and lower esophageal involvement showed borderline associations with stent dysfunction; however, none demonstrated significant effect modification by stent type after adjustment (Table 6).

**Table 6:** Adjusted Fine-Gray subdistribution hazard models for stent dysfunction by stent type, with mortality treated as a competing risk

	pcSEMS		fcSEMS		Interaction (pcSEMS ref.)	
	sHR (95% CI)	*P*	sHR (95% CI)	*P*	sHR_int_ (95% CI)	*P*_int_
Age, years	1.029 (0.981–1.078)	0.24	1.029 (0.996–1.062)	0.08	1.004 (0.948–1.064)	0.89
Sex, male	0.889 (0.234–3.371)	0.86	1.264 (0.512–3.120)	0.61	1.278 (0.248–6.599)	0.77
BMI, kg/m^2^	1.007 (0.938–1.082)	0.84	1.091 (0.997–1.193)	0.06	1.089 (0.976–1.215)	0.13
ASA class ≥ III	1.261 (0.418–3.804)	0.68	1.682 (0.753–3.757)	0.21	1.367 (0.359–5.212)	0.65
Cardiac comorbidity	1.362 (0.504–3.680)	0.54	1.749 (0.699–4.375)	0.23	1.252 (0.357–4.392)	0.73
Diabetes mellitus	1.053 (0.279–3.977)	0.94	1.644 (0.688–3.930)	0.26	1.519 (0.360–6.400)	0.57
Adenocarcinoma	2.092 (0.608–7.190)	0.24	1.074 (0.518–2.223)	0.85	0.705 (0.210–2.373)	0.57
Squamous cell carcinoma	1.010 (0.316–3.226)	0.98	0.557 (0.265–1.168)	0.12	0.420 (0.113–1.561)	0.20
Other malignancies	0.407 (0.107–1.551)	0.19	6.525 (2.507–16.980)	< 0.01	14.105 (2.076–95.819)	< 0.01
Prior chemoradiotherapy	1.251 (0.354–4.419)	0.73	0.348 (0.062–1.949)	0.23	0.260 (0.032–2.124)	0.21
Ogilvie dysphagia score	0.982 (0.517–1.863)	0.95	0.350 (0.105–1.160)	0.09	0.339 (0.097–1.187)	0.09
Passed endoscopically	1.091 (0.326–3.652)	0.89	1.512 (0.708–3.226)	0.29	1.862 (0.494–7.014)	0.36
Distance from incisors, cm	0.991 (0.889–1.104)	0.87	1.043 (0.986–1.103)	0.14	1.062 (0.954–1.183)	0.27
Upper esophagus	1.769 (0.308–10.161)	0.52	0.261 (0.025–2.780)	0.27	0.141 (0.010–2.062)	0.15
Middle esophagus	0.890 (0.282–2.807)	0.84	0.594 (0.220–1.609)	0.31	0.531 (0.131–2.145)	0.37
Lower esophagus	0.891 (0.271–2.928)	0.85	2.333 (0.895–6.077)	0.08	3.104 (0.809–11.902)	0.10
GEJ involved	3.569 (0.568–22.419)	0.18	1.600 (0.770–3.327)	0.21	0.867 (0.254–2.962)	0.82
Stricture length, mm	1.005 (0.990–1.021)	0.53	1.011 (0.995–1.026)	0.17	1.004 (0.984–1.024)	0.73
Stricture diameter, mm	0.987 (0.756–1.290)	0.92	1.206 (1.005–1.447)	0.04	1.222 (0.894–1.671)	0.21
Stent length, mm	0.989 (0.956–1.022)	0.50	1.001 (0.985–1.017)	0.91	1.004 (0.970–1.038)	0.84
Stent diameter, mm	0.897 (0.688–1.170)	0.42	1.101 (0.873–1.390)	0.41	1.224 (0.881–1.700)	0.23

Abbreviations: ASA, American Society of Anesthesiologists; BMI, body mass index; fcSEMS, fully covered self-expanding metal stent; GEJ, gastroesophageal junction; pcSEMS, partially covered self-expanding metal stent; *P*_int_, p value for interaction; *ref.*, reference; sHR, subdistribution hazard ratio; sHR_int_, interaction subdistribution hazard ratio

Model adjusted for age, sex, BMI, ASA class, and prior chemoradiotherapy

### Sensitivity analysis according to tumor etiology

To examine whether the observed associations were driven by etiologic case mix, analyses were repeated after excluding strictures caused by other malignancies (*n* = 39) and stratified by AC and SCC. Within both histological subgroups, fcSEMS were not independently associated with stent dysfunction after adjustment for the same covariates (AC: adjusted sHR 0.92; 95% CI 0.36–2.36; *p* = 0.86; SCC: adjusted sHR 0.59; 95% CI 0.19–1.83; *p* = 0.36), supporting the marked contribution of other malignancies to the overall stent type-specific effect. Stricture diameter was not significantly associated with dysfunction after stratification by tumor etiology, although the point estimate remained higher in AC (adjusted sHR per mm 1.21; 95% CI 0.80–1.83; *p* = 0.37) than in SCC (adjusted sHR per mm 1.05; 95% CI 0.88–1.25; *p* = 0.63). However, the smaller sample size in this subanalysis limited statistical power, and clinically relevant associations therefore cannot be excluded. Importantly, lower esophageal location remained independently associated with dysfunction in SCC (adjusted sHR 3.13; 95% CI 1.06–9.25; *p* = 0.03), indicating that distal stricture anatomy remained clinically relevant after restriction by tumor etiology.

## Discussion

In this multicenter retrospective study, we compared fully versus partially covered SEMS for the palliation of malignant esophageal strictures and investigated whether determinants of stent dysfunction differed by stent type, while accounting for death as a competing event.

In multivariable analyses, larger stricture diameter and strictures caused by other malignancies independently predicted stent dysfunction in the fcSEMS group, whereas lower esophageal location showed a borderline association. Formal interaction testing demonstrated significant effect modification by stent type for other malignancies and a nonsignificant trend toward effect modification for lower esophageal location. In addition, selected procedural and patient-reported variables may help estimate the risk of stent dysfunction at the index procedure.

Stent dysfunction is considered the leading cause of recurrent dysphagia after SEMS placement for malignant esophageal strictures [7, 8]. In our cohort, dysfunction occurred in 30% of patients, most commonly due to migration (21%). Stent dysfunction was more frequent with fcSEMS than with pcSEMS (36.5% vs. 22.8%), primarily driven by a higher rate of stent migration (31.3% vs. 10.9%). A large multicenter retrospective study similarly reported higher migration rates with fcSEMS than with pcSEMS [10].

Regarding stent occlusion, a single-center retrospective study reported higher rates of overgrowth with pcSEMS compared with fcSEMS [18]. In contrast, a prospective multicenter trial found no significant differences in stent-related adverse events, including tumor overgrowth, between the two stent types [9]. Consistent with the latter, we observed no significant difference in the incidence of stent dysfunction due to overgrowth or ingrowth between fcSEMS and pcSEMS.

We observed etiology-related differences in stent dysfunction, suggesting that tumor type may influence the risk of dysfunction. Esophageal SCC was associated with a lower observed risk than AC and strictures caused by other malignancies. However, estimates within the fcSEMS group should be interpreted with caution because of the small number of cases and limited statistical power. The literature on the relationship between tumor etiology and stent dysfunction remains limited. In one study, AC was independently associated with a lower risk of pain; however, etiology-specific dysfunction rates were not reported [8]. Similarly, another cohort analysis found no significant effect of histopathological diagnosis on overall complication rates [19]. Importantly, prior studies have not evaluated histology in interaction with stent covering.

Stricture location emerged as another clinically relevant determinant of stent dysfunction. Lower esophageal involvement showed a borderline association with increased risk in the adjusted model, whereas distance from the incisors was not significantly associated with dysfunction. These findings are only partly consistent with previous studies, some of which found no association between stricture location and stent dysfunction [10, 20]. However, they support reports of greater stent instability in distal esophageal strictures [21, 22]. This may reflect increased mechanical stress from diaphragmatic motion and the transition from the tubular esophagus to the saccular stomach [23, 24], which may compromise stent anchoring and promote migration [25, 26].

Endoscopic traversability before SEMS placement may provide a clinically useful indicator of subsequent stent dysfunction. Pediatric gastroscope use was associated with a lower subdistribution hazard in both stent groups. However, the low number of dysfunction events produced unstable estimates and precluded reliable interpretation of the interaction with stent type. Because pediatric gastroscopes are generally used when a standard adult gastroscope (≥ 9 mm) cannot traverse the stricture, their use may serve as a surrogate marker of a tighter residual lumen. This interpretation is consistent with a previous study showing that preserved luminal patency, reflected by the passage of a standard gastroscope, independently predicted stent migration [10]. It is further supported by the association between larger stricture diameter and increased dysfunction, with an approximately 20% increase in the subdistribution hazard per 1-mm increment in the fcSEMS group. A wider residual lumen may provide less radial resistance and weaker mechanical anchoring, thereby facilitating stent migration.

Strengths of this study include its multicenter design, predefined comprehensive data collection, and use of real-world data reflecting routine clinical practice. Methodologically, the analysis accounted for death as a competing event through cumulative incidence estimates at predefined time points and multivariable Fine-Gray subdistribution hazard models. Formal interaction testing further enabled the assessment of stent type-specific risk patterns beyond descriptive subgroup comparisons.

However, several limitations should be acknowledged. First, the retrospective design carries an inherent risk of selection bias, although the clinical course and follow-up were generally well documented. Second, stent allocation was not randomized and may have been influenced by local device availability and clinical judgment. Third, residual confounding related to center-specific stent availability, clinical practice, and operator preferences cannot be excluded. Fourth, baseline differences between the stent groups, including comorbidity burden and tumor characteristics, may have influenced the observed outcomes despite multivariable adjustment. Fifth, due to the retrospective design, standardized patient-reported outcome measures were not available for all patients. Finally, some subgroup analyses were limited by small sample sizes, resulting in potentially unstable estimates that should be interpreted with caution. In particular, findings in the “other malignancies” subgroup should be considered exploratory and hypothesis-generating.

In conclusion, predictors of stent dysfunction varied according to stent type, with tumor etiology as a key contributor to differences between pcSEMS and fcSEMS. Stricture diameter was independently associated with stent dysfunction in the fcSEMS group, while lower esophageal location showed a borderline association. However, neither factor demonstrated significant effect modification by stent type compared with pcSEMS. Together, these findings support a risk-stratified approach to stent selection that considers tumor- and stricture-related characteristics alongside stent type. Validation in independent prospective cohorts is warranted before routine clinical application. Future research should also focus on refinements in stent technology, including improved anti-migration features and segmented or adaptive designs. Patient-reported outcomes should receive greater emphasis, as the primary aim of stent therapy in malignant esophageal strictures is to relieve dysphagia and improve quality of life.

## Data Availability

The datasets generated and/or analyzed during the current study are not publicly available due to institutional and data protection restrictions but are available from the corresponding author on reasonable request.

## Abbreviations

AC: Esophageal adenocarcinoma
AE: Adverse event
ASA: American Society of Anesthesiologists
ASGE: American Society for Gastrointestinal Endoscopy
BMI: Body mass index
CI: Confidence interval
fcSEMS: Fully covered self-expanding metal stent
GEJ: Gastroesophageal junction
INR: International normalized ratio
IQR: Interquartile range
KPS: Karnofsky Performance Status
pcSEMS: Partially covered self-expanding metal stent
SCC: Esophageal squamous cell carcinoma
SD: Standard deviation
SEMS: Self-expanding metal stent
sHR: Subdistribution hazard ratio
